# Clinical study of recurrent mild encephalitis/encephalopathy with a reversible splenial lesion in two cases

**DOI:** 10.1186/s12887-021-03097-x

**Published:** 2022-01-03

**Authors:** Jiao Xue, Chongfeng Duan, Guizhi Xu, Ying Zhang, Zhenfeng Song, Zhi Yi, Chengqing Yang, Fei Li, Kaixuan Liu, Hongshan Zhao, Xuejun Liu

**Affiliations:** 1 Department of Pediatric Neurology, the Affiliated Hospital of Qingdao University, Qingdao, 266000 Shandong China; 2 Department of Radiology, the Affiliated Hospital of Qingdao University, Qingdao, No. 1677 Wutaishan Road, Qingdao, Shandong 266000 PR China; 3 Department of Radiology, Zhucheng People Hospital, Weifang, 262200 Shandong China; 4 Department of Anesthesiology, the Affiliated Hospital of Qingdao University, Qingdao, 266000 Shandong China

**Keywords:** Child, Recurrent, MERS

## Abstract

**Background:**

Mild encephalitis/encephalopathy with a reversible splenial lesion (MERS) has been reported worldwidely. However, the data about recurrent cases is limited. We aimed to analyze the clinical and radiographic features of recurrent MERS, and its possible mechanisms.

**Case presentation:**

Two patients with clinically recurrent MERS were reported here, exhibiting neurological symptoms such as limbs weakness and numbness, stand/walk unsteadily, slurred speech and irritability, and typical lesions in the corpus callosum and white matter. One of them experienced another four episodes with a similar clinical course and magnetic resonance imaging findings over a period of 10 years. The Na levels in the present two patients were normal.

**Discussion and conclusion:**

Combined with the patients reported previously, recurrence could be seen in both MERS type 1 and type 2 patients, from two to multiple times, with the latter possibly more common. It suggested that some genetic factors might be involved in MERS, especially for MERS type 2 or familial MERS.

## Background

Mild encephalitis/encephalopathy with a reversible splenial lesion (MERS) is a clinico-radiological syndrome first described by Tada et al. [1] at 2004, characterized by transient lesions in the corpus callosum on magnetic resonance imaging (MRI) and mild encephalopathy following prodromal symptoms such as fever, cough, vomiting and/or diarrhea, which usually recover within a month [2]. It was further proposed to be classified into MERS type 1, with an isolated lesion in the splenium of the corpus callosum, and MERS type 2, with an extensive white matter and/or entire callosal lesions [3]. The pathogenesis of MERS is still not fully understood. Many studies of MERS have been reported in recent years, involving clinical and imaging features, as well as triggering factors, etc. [2, 4–6]. However, research on recurrent MERS is extremely rare. Here, we reported the clinico-radiological features of 2 cases with recurrent MERS, and discussed the possible pathogenic mechanisms.

## Case presentation

### Case 1

**Fig. 1:**
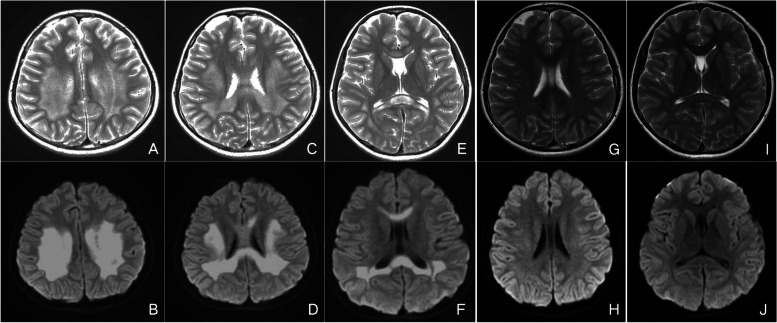
Cranial MRI of case 1 in 2016. Cranial MRI on day 2 showed lesions in the entire corpus callosum and diffuse white matter with moderate hyper intensity on T2-weighted images (**A**,**C**,**E**) and marked hyperintensity on DWI (**B**,**D**,**F**). Follow-up MRI on day 12 showed resolution of the lesions on T2 (**G**,**I**) and DWI (**H**,**J**)

In April 2016, a previously healthy 11-year-old boy was admitted to our hospital due to intermittent right upper limb weakness, numbness in the right corner of the mouth, irritability and then anepia and stand unsteadily sometimes, lasting for several hours. There was no family history or past history of neurological disorders, including epilepsy and developmental retardation. The results of neurological examination were unremarkable. Blood examinations revealed a normal white blood cell count (5.80 × 10^9/L) and C-reactive protein level (0.90 mg/L), and normal levels of ammonia, glucose and Na (138 mmol/l). Anti-nuclear antibody and anti-neutrophils cytoplasmic antibody were normal also. Cerebrospinal fluid (CSF) examinations showed normal cell counts, and protein and glucose levels. Cranial MRI on day 2 revealed lesions in the entire corpus callosum and diffuse white matter with moderate hyper intensity on T2-weighted images, and marked hyperintensity on diffusion-weighted images (DWI) (Fig. 1A-F). A diagnosis of MERS type 2 was considered according to the clinical and radiological features. He was treated with intravenous methylprednisone and antiviral therapy; his clinical manifestations gradually improved, and he had completely recovered by day 5. Follow-up MRI on day 12 showed resolution of the lesions on T2 and DWI (Fig. 1G-J).

**Fig. 2:**
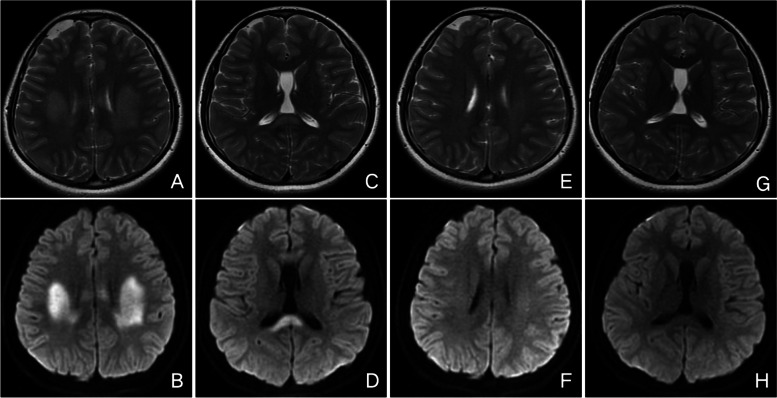
Cranial MRI of case 1 in 2018. Initial cranial MRI showed lesions in the entire corpus callosum and diffuse white matter on T2-weighted images (**A**,**C**) and DWI (**B**,**D**). Follow-up MRI on day 18 showed resolution of the lesions on T2 (**E**,**G**) and DWI (**F**,**H**)

In August 2018, he was admitted to our hospital again because of right limb weakness and numbness with right face numbness and a feeble voice, and then left limb weakness and numbness. The results of neurological examination were unremarkable. Blood examinations showed no special abnormalities. Serum Na (142 mmol/l) was normal. Electroencephalography (EEG) was also normal with no epileptic discharges or high voltage slow waves. Cranial MRI showed similar lesions in the entire corpus callosum and diffuse white matter as before (Fig. 2A-D). He was considered as recurrent MERS type 2 and treated with antiviral drug and mecobalamine to nourish the nerve. His clinical manifestations completely recovered by day 3. Then a follow-up MRI on day 18 showed resolution of the lesions (Fig. 2E-H).

### Case 2

In August 2010, a previously healthy 5-year-old boy was admitted to our hospital for slurred speech, limbs weakness, walk unsteadily with tumble sometimes. He had a prodromal infection of cough, fever, vomit and headache 2 weeks before this event. There was no family history or past history of neurological disorders. He had received antibiotics (ampicillin, cefuroxime and azithromycin) and Ibuprofen before admission. On admission, the muscle strength and tone of the limbs were normal. The results of neurological examination showed mild positive of Kernig sign, Babinski sign and Chaddock sign. Blood examinations revealed a mild elevated white blood cell count (11.08 × 10^9/L) and normal C-reactive protein (0.49 mg/L), glucose and Na (139 mmol/l) level. Cranial MRI revealed lesions in the splenium of the corpus callosum and diffuse white matter including semi-ovoid center and periventricular with moderate hyper intensity on T2-weighted images, and marked hyperintensity on DWI (Figures unavailable). Cervical, thoracic and lumbar MRI were normal. MERS has not widely recognized at that time. He was diagnosed as encephalitis post-infected and was treated with intravenous methylprednisone, anti-infective therapy (ganciclovir and cefepime) and mannitol. His clinical manifestations gradually improved, and he had completely recovered by day 4, with a negative neurological examination. Follow-up MRI on day 15 showed a resolution of the lesion in the splenium of the corpus callosum and diminished lesion in periventricular matter with moderate hyper intensity on T2-weighted images but normal on DWI (Figures unavailable).

**Fig. 3:**
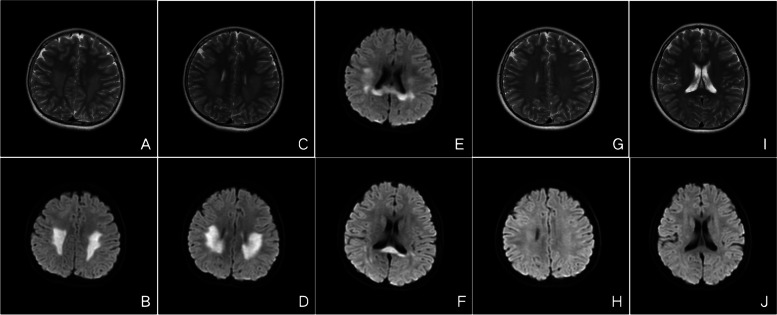
Cranial MRI of case 2 in 2015. Initial cranial MRI showed lesions in the entire corpus callosum and diffuse white matter on T2-weighted images (**A**,**C**) and DWI (**B**,**D**-**F**). Follow-up MRI showed resolution of the lesions on T2 (**G**,**I**) and DWI (**H**,**J**) after seven weeks

**Fig. 4:**
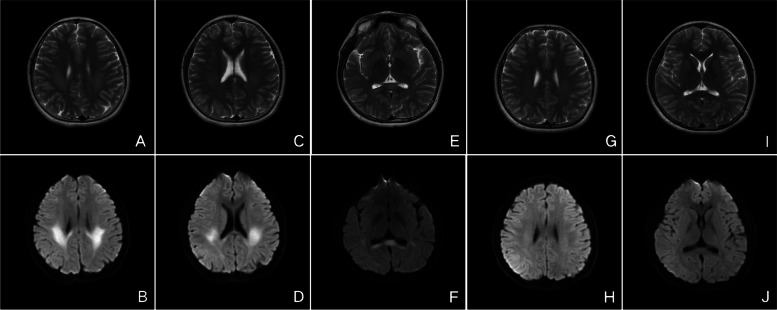
Cranial MRI of case 2 in 2017. Initial cranial MRI showed lesions in the entire corpus callosum and diffuse white matter T2-weighted images (**A**,**B**,**E**) and marked hyperintensity on DWI (**B**,**D**,**F**). Follow-up MRI showed resolution of the lesions on T2 (**G**,**I**) and DWI (**H**,**J**) after 3 months

And then he experienced four episodes with a similar clinical course and MR imaging findings over a period of 10 years (in 2012, 2015, 2017 and 2020 yeas, respectively) (Fig. 3A-F; Fig. 4A-F), and was considered as recurrent MERS type 2. The clinical manifestations disappeared within several days under the treatment of intravenous methylprednisone. And the cranial MRI after several weeks showed resolution of the lesions completely (Fig. 3G-J; Fig. 4G-J). He had normal psychomotor development without any neurological sequelae.

## Discussion and conclusion

MERS is a subtype of acute encephalopathy state with characteristic diagnostic MRI findings suggesting myelin vacuolization [1, 7]. Although a great number of cases has been reported worldwidely, the data about recurrent MERS is limited to a really few case report. In this report, we described two cases with recurrent MERS type 2, and one of them had multiple relapses. Although the cranial MRI of one patient (case 2) was not normal completely at discharge, it was reasonable to speculate that the lesion would disappear with time given the significant reduction in lesion scope and signal intensity, as well as the followed similar episodes.

After browsing in Wanfang, CNKI and PubMed database, as well as searching the references for related published articles, we found only several cases with recurrent MERS in literatures. Dong et al. [8] reported a girl who experienced repeated episodes of convulsions at 19 and 21 months of age, following cough and vomit respectively. The serum Na was normal. After anti-infective and glucocorticoid treatment, clinical symptoms disappeared within a few days. Both cranial MRI revealed a lesion in the splenium of the corpus callosum at first and disappeared on day 7 and day 8, respectively. And a diagnosis of MERS type 1 was confirmed. Duan et al. [9] reported a boy with recurrent MERS type 2, which was included in our study here (case 1). Moreover, Imamura et al. [10] mentioned that a 5-year-old girl who experienced MERS three times and Zhang et al. [11] described a child who experienced MERS two times. However, neither specific clinical nor imaging description of these two patients was offered. Besides, Shigeno et al. [12] reported a rare case of MERS associated with transient ischemic attact (TIA)-like symptoms. He presented with transient left hemiparesis and dysarthria at 9 years old and was diagnosed with MERS type 2 due to the typical clinical course and MRI findings. Then he had experienced a similar symptom at 13 years old and a diagnosis of recurrence of MERS type 2 was considered. And then he experienced eight TIA-like episodes with a similar clinical course and MRI findings over a period of 6 years. It was worth noting that one of our patients (case 1) was mainly characterized by unilateral limb weakness and numbness, which was similar to the TIA-like symptoms described by Shigeno et al. [12]. If so, the possibility of multiple recurrences cannot be ruled out and further follow-up is needed. Combined with the patients reported previously and our two patients, it suggests that both MERS type 1 and MERS type 2 are likely to relapse, one or even more times. It seems that patients with type 2 may be more likely to relapse, but in view of the small number of cases, no firm conclusions can be drawn yet.

The exact pathomechanism of MERS remains unclear. PPossible mechanisms include intramyelinic edema, a transient inflammatory infiltrate, and interstitial edema in tightly packed fibers [1, 2]. Laboratory evaluation revealed that hyponatremia is common in patients with MERS [13]. Hypotonic hyponatremia causes water to enter the brain, resulting in cerebral edema. The Na levels in the present two patients, as well as in the patient reported by Dong et al. [8], however, were normal, which suggested that the pathophysiology of the recurrent MERS might be different from that of sporadic MERS.

Kurahashi et al. [14] reported a family with recurrent encephalopathy spanning over three generations, which presented with extensive but reversible cerebral myelin vacuolization and neurological symptoms similar MERS. And a heterozygous c.1208A > G (p.Gln403Arg) in the myelin regulatory factor (MYRF) gene was identified [10, 14]. As a transcriptional regulator, MYRF is significant for oligodendrocyte differentiation and myelin maintenance [15]. Therefore, functional deficiency of MYRF may be causally related to extensive myelin vacuolation [16]. Although Kurahashi et al. [14] suggested that MYRF variant cases and sporadic MERS cases might be different conditions. They also mentioned that the central nervous system (CNS) symptoms of MYRF variant cases were actually indistinguishable from MERS, and MRI features involving widespread white matter and the entire corpus callosum were also very similar to the MERS type 2, which suggested that MYRF variant cases might be a more extensive form of MERS. Meanwhile, the MYRF variant was also identified in another family that they partially described as familial MERS-like features (MERS type 2?) [10]. Therefore, it is reasonable to believe that some genetic factors might be involved in MERS, especially for MERS type 2 or familial MERS.

In conclusion, recurrence could be seen in both MERS type 1 and type 2 patients, from two to multiple times, with the latter possibly more common. Recurrent and/or familial cases with a clinical diagnosis of MERS suggest the presence of genetic factors. Further clinical, radiological and genetic studies are needed for a definite conclusion.

## Data Availability

Datasets used and/or analysed during the current study are available from the corresponding author on reasonable request.

## Abbreviations

MERS: Mild encephalitis/encephalopathy with a reversible splenial lesion
MRI: Magnetic resonance imaging
CSF: Cerebrospinal fluid
DWI: Diffusion-weighted images
EEG: Electroencephalography
TIA: Transient ischemic attact
MYRF: Myelin regulatory factor
